# Complete probabilistic analysis of RNA shapes

**DOI:** 10.1186/1741-7007-4-5

**Published:** 2006-02-15

**Authors:** Björn Voß, Robert Giegerich, Marc Rehmsmeier

**Affiliations:** 1 Faculty of Technology, Bielefeld University, 33594 Bielefeld, Germany; 2 Institute of Biology II, Experimental Bioinformatics, Freiburg University, Schaenzlestr. 1, 79104 Freiburg, Germany; 3 Faculty of Technology, Bielefeld University, 33594 Bielefeld, Germany; 4 Center for Biotechnology (CeBiTec), Bielefeld University, 33594 Bielefeld, Germany

## Abstract

**Background:**

Soon after the first algorithms for RNA folding became available, it was recognised that the prediction of only one energetically optimal structure is insufficient to achieve reliable results. An in-depth analysis of the folding space as a whole appeared necessary to deduce the structural properties of a given RNA molecule reliably. Folding space analysis comprises various methods such as suboptimal folding, computation of base pair probabilities, sampling procedures and abstract shape analysis. Common to many approaches is the idea of partitioning the folding space into classes of structures, for which certain properties can be derived.

**Results:**

In this paper we extend the approach of abstract shape analysis. We show how to compute the accumulated probabilities of all structures that share the same shape. While this implies a complete (non-heuristic) analysis of the folding space, the computational effort depends only on the size of the shape space, which is much smaller. This approach has been integrated into the tool RNAshapes, and we apply it to various RNAs.

**Conclusion:**

Analyses of conformational switches show the existence of two shapes with probabilities approximately $\frac{2}{3}$ vs. $\frac{1}{3}$, whereas the analysis of a microRNA precursor reveals one shape with a probability near to 1.0. Furthermore, it is shown that a shape can outperform an energetically more favourable one by achieving a higher probability. From these results, and the fact that we use a complete and exact analysis of the folding space, we conclude that this approach opens up new and promising routes for investigating and understanding RNA secondary structure.

## Background

RNA secondary structure analysis is a common task in research on RNA and its manyfold functions. The first algorithm capable of computing the structure with minimum free energy (MFE) based on the *nearest neighbour energy model *was introduced in [1]. It was capable of calculating the MFE-structure only, and gave valuable results for short sequences. Nevertheless, it was recognised that although predicted RNA secondary structures contain, on average, 73% of known base pairs for RNA sequences divided into domains of less than 700 nucleotides [2], the predicted structures are sometimes quite different from the secondary structures obtained by comparative sequence analysis. After two decades of refined measurements of thermodynamic parameters, the problem persists [3], and the limited credibility of the MFE-structure is attributed to intrinsic properties of the folding space, such as its partioning into families of similar structures and the kinetics of folding [4,5].

The state of an RNA molecule must be seen as a Boltzmann ensemble of structures, some very similar, some quite distinct. The challenge of folding space analysis is to determine whether there is some family of structures in this ensemble that is internally similar, distinct from the rest, and collectively dominates the probabilities of all other families. The dominating family, if any, should be the biologically relevant one. For this reason, it is of interest to include suboptimal solutions in the process of structure elucidation. In [6] Zuker introduced an extended version of his algorithm, which was also capable of predicting certain suboptimal structures. This allows a researcher to check different predictions for correspondence to experimental results. The most recent version of the algorithm [2,7] is implemented in the MFOLD package [8].

A drawback of the Zuker algorithm is the use of heuristic filters to circumvent the repeated output of the same structure. These filters remove not only redundant structures but also similar structures. This is desirable from a human observer's point of view, but precludes a probabilistic analysis. In [9], an algorithm was introduced allowing for non-redundant and complete suboptimal folding, which is implemented in the tool RNAsubopt from the Vienna RNA package [10]. It is designed to compute rigorously all structures within a given energy range and is guaranteed not to miss any structure that is feasible with respect to the nearest neighbour energy model. The major advantage of this approach is that it gives access to all suboptimal structures, i.e. the complete folding space of an RNA sequence. However, as the number of structures is exponentially related to sequence length [11], this method produces a large number of structures, which are laborious to analyse.

The free energies of RNA structures can be imagined as a rough landscape over the folding space. The folding space is described by the notion of neighbourhood, which in the case of RNA secondary structure is the difference in exactly one base pair. A structure having only neighbours with higher free energy is a *local minimum *and forms the bottom of a *valley*. All structures that can be reached by neighbour moves (opening or closing of a base pair) while increasing the energy form a valley in the landscape and can be seen as a *family *of structures. A structure having neighbours in more than one valley is referred to as a *saddle point *in the landscape. Rephrasing our challenge in this alpine terminology, the task is not only to find the lowest point overall, but to relate the depths of valleys to their population sizes, and to determine the family of which members are most likely to be encountered when this landscape is explored. The first method (even prior to RNAsubopt) for analysing the complete folding space in order to assess the relevance of a secondary structure was introduced by McCaskill in [12]. The author makes use of the partition function to address this property. In general, the partition function provides a measure of the total number of states (structures) weighted by their individual energies at a particular temperature. For an RNA sequence and the set *S *of all possible structures for this sequence, it is defined as follows:

$Q=\sum_{j\in S}e^{\frac{-E_{j}}{RT}}\;\left(1\right)$

where *E*_*j *_is the energy of structure *j, R *the universal gas constant (0.00198717 kcal/K) and *T *the temperature in Kelvin. In words, this is the sum of the Boltzmann weighted energies of all structures. The probability *P *of a particular secondary structure *x *∈ *S *is defined as:

$P(x)=e^{\frac{-E_{x}}{RT}}/Q\;\left(2\right)$

where *E*_*x *_is the energy of structure *x *in kcal/mol. Intrinsic to this approach is that the probability is proportional to the (Boltzmann weighted) energy of a structure. Hence, this approach provides no further information on structural relevance. No individual structure can have a higher probability than a structure with lower free energy, and the MFE-structure is always the most probable one; albeit with an individual probability that is often very close to zero. This problem has already been stated in [12], and the author also provides a means to alleviate it. Instead of computing the probability of a complete structure, the probabilities of atomic structural elements, i.e. base pairs, are computed. Displaying these in a matrix, as squares with area proportional to the probability, results in the so called "dot plot" for base pairing probabilities. This visualisation shows all possible base pairs and allows for the detection of alternative structures with high probability.

The partition function cannot only be used to calculate the probabilities of individual structures or base pairs. In [4], Ding and Lawrence introduced a statistical sampling algorithm that is implemented in the tool SFOLD. In each step of the recursive backtracing procedure, base pairs and the structural elements they belong to are sampled according to their probabilities, obtained from the partition function. Features of the sampling procedure are that each run is likely to produce a different sample and that the same structure can be sampled multiple times, where the MFE-structure is the most frequent structure, as it has the highest probability. Nevertheless, the MFE-structure is not guaranteed to be present in the sample, especially for long sequences. The authors showed that sampling the folding space of the Spliced Leader of *Leptomonas collosoma *could give structures from two families. These two families, which were defined by manual alignment of the sampled structures, correspond to the alternating structures of this conformational switch. This improves over a non-probabilistic sampling procedure yielding simlar results [13,14].

Another tool analysing the complete folding space of an RNA, or part thereof, is barriers [15] by Flamm *et al. *It is designed to find local minima and saddle points connecting these, and in addition it generates the so-called "barrier tree" as a visualisation of the landscape. In the barrier tree, the local minima are leaves and saddle points are nodes connecting either two local minima, a local minimum and a saddle point, or two saddle points. The length of an edge corresponds to the energy difference between the connected elements.

Common to all approaches is the attempt to partition the folding space into structural families and to derive features of each such family. For the partitioning, Zuker uses structural similarity based on a distance measure, McCaskill base pairs, Ding and Lawrence similarity of sampled structures and Flamm *et al. *the affiliation to the same valley. One problem persists: exhaustive enumeration is slow, while sampling cannot clearly designate a dominating family.

In principle, the local minima in the folding space neighbourhoods can be taken as representatives of all the families. Unfortunately, no algorithm has been found that computes these representatives directly, i.e. without explicit enumeration of all individual structures. From a more macroscopic point of view, the notion of neighbourhood based on base pair opening and closing seems too low-level anyway: two alternative structures may both have (say) a cloverleaf shape, but not share a single base pair, and hence belong to different families. Having the same shape is therefore a stronger abstraction. It retains adjacency and nesting of stacks and hairpins. It gives us the option of regarding or disregarding the presence of bulges. And it completely abstracts from individual base pairs and their location in the sequence. This idea has been formalized in the approach of abstract shape analysis of RNA [16]. Each shape is a distinct class of structures, which has a representative structure, *shrep *for short, of minimal free energy within the shape. These *shreps *can be computed directly – avoiding the burden of exhaustive enumeration of individual structures. It has been shown that computing the *k *lowest-energy *shreps *provides useful information. Abstract shape analysis, as described in [16], can also provide precise accounts of the number of structures within each shape – but perhaps surprisingly, it does not provide the overall probability of a shape. This is the classical challenge formulated above, and a solution based on the concept of shapes will be described in this contribution.

### Outline of probabilistic shape analysis

Complete probabilistic shape analysis computes, for each shape, the probability sum of the structures within that shape. While this goal is simply stated, it is more difficult and computationally more costly to achieve than simple shape analysis. Our presentation is organised as follows:

We first show how to compute the shapes and Boltzmann-weighted energies of individual structures, and, by analogous means, the partition function. We then combine these calculations by a programming technique called classified dynamic programming. It allows to accumulate the Boltzmann-weighted energies of all structures by shape. We then study the algorithmic efficiency of this calculation, where we find that it avoids exponential relationship to the number of structures, but is exponentially related to the number of shapes. In sharp contrast to the number of structures, the number of shapes is typically small enough to make the approach practical. Finally, we report on applications of complete probabilistic shape analysis to several types of RNA, and discuss the results.

## Results

In the first three subsections, we explain the mathematical model underlying our new type of analysis. (Algorithmic details and efficiency concerns are deferred to the Methods section.) Subsequently, we report on the findings of various applications of the method.

### Modelling the folding space

RNA secondary structures can be represented as strings, base pair lists, graphics, and in many other forms. When they are to be analysed under different objective functions, and when pseudoknots are not involved, RNA secondary structures are most conveniently represented as trees. Trees allow the pattern of helix adjacency and nesting that characterises a secondary structure to be represented naturally. The tree-like representations presented here will not subsequently be computed. Instead, various secondary structure features will be computed (such as their free energy, shape, string representation, etc.), and the tree-like structure representations will serve as a common model for the precise and uniform definition of these derived features.

Our structure representations are rooted, ordered, labelled trees. On their leaf nodes, in left to right order, the labels spell out the primary sequence, built from nucleotides A, C, G and U. The inner nodes of the tree are labelled by operators related to the structural features of RNA: single stranded regions (SS), hairpin loops (HL), stacked base pairs that form stacking regions (SR), 5' and 3' bulges (BL and BR), internal loops (IL) and multiloops (ML). Multiloops comprise a closing base pair and a list of adjacent (AD) structure elements inside. For mathematical completeness, we also need operator E denoting an empty list of adjacent structures.

Figure 1.1 gives an example tree. It shows the representation of a small hairpin embedded in two single strands. In the more familiar dot-bracket notation, its representation would be ". . ( ( ( ( . ( . . . . ) . ) ) ) ) .". While the string representation is easier for us to read, the trees are mathematically more convenient. Each operator can also be seen as a function symbol, taking a fixed number of arguments of fixed types. For example, the BL operator accepts a (closing) pair of bases, say (*a*, *b*), a single stranded region *l*, and a closed substructure *x*. We can write the formula *BL*(*a*, *l*, *x*, *b*), which is equivalent to the tree. This interpretation of operators as function symbols that compose structures is summarized in Table 1. Our example structure is shown as a formula in Figure 1.2.

**Table 1:** Secondary structure operators. Operators build terms by application to (sub-)terms. Operators can be interpreted in different ways with algebras, such as the Boltzmann-weighted energy algebra. In this case, terms evaluate to real numbers. Interpreting operators as mere symbols leads to symbolic terms that represent structures, (cf. also Figure 1)

operator	description
SS(l)	single-stranded region l
HL(a,l,b)	hairpin loop with single stranded region l, closed by basepair (a,b)
SR(a,x,b)	stacking region, closed by basepair (a,b); x is a closed structure
BL(a,l,x,b)	bulge left with single stranded region l, closed by basepair (a,b); x is a closed structure
BR(a,x,l,b)	bulge right with single stranded region l, closed by basepair (a,b); x is a closed structure
IL(a,l,x,l',b)	internal loop with single stranded regions l and l', closed by basepair (a,b); x is a closed structure
ML(a,c,b)	multi-loop, closed by basepair (a,b)
AD(x,c)	list of adjacent structures; x is a structure, c a (possibly empty) list of adjacent structures
E	empty list of adjacent structures

It is important to note that not all such trees or formulas represent legal structures. For example, *ML*('*C*', *HL*('*A*', "*CCC*", '*U*'), '*G*') is valid as a formula – every operator has the right number and type of arguments – but not valid as a structure, since the notion of a multiloop implies that there are at least two closed substructures inside. Rules for building trees that are valid structures can be given in the form of a tree grammar. The mathematical appeal of a tree grammar is that, besides providing a precise definition of the folding space associated with a given RNA molecule, it can automatically be converted into a parsing algorithm, based on dynamic programming, that evaluates this folding space. For example, it can find the minimal free energy structure, or derive any other type of information that can be described in the systematic fashion introduced below.

A tree grammar for RNA secondary structures is shown in Table 2. We use an ASCII representation of grammar rules, which is both easy to read *and *suitable for algorithm generation. A clause such as

**Table 2:** Basic secondary structure grammar. This grammar is a simplified version, included for illustrative purposes. The grammar that is actually used for calculating shape probabilities is larger, owing to the requirement to be unambiguous; see the discussion in paragraph "A non-ambiguous grammar with correct dangles" and Table 6. Part a) shows the grammar in its algebraic form. | | | signifies alternative right-hand sides of productions, ... h the application of choice function h, ^~~~ ^juxtaposition of terms. <<< denotes application of the operator to its left-hand side to the arguments of its right-hand side. Operators are as in Table 1, plus ul(x) as an abbreviation for ad(x,e), str for structures, and blk for blocks. The axiom of the grammar is struct. Part b) shows the same grammar in EBNF notation, naturally without the operators to be applied.

a)
struct = str <<< comps |||
str <<< singlestrand |||
str <<< (e <<< empty) ... h
block = ad <<< singlestrand ^~~~ ^closed ... h
comps = ad <<< block ^~~~ ^comps |||
block
ad <<< block ^~~~ ^singlestrand ... h
singlestrand = ss <<< region
closed = (hl <<< base ^~~~ ^region3 ^~~~ ^base |||
sp <<< base ^~~~ ^closed ^~~~ ^base |||
sr <<< base ^~~~ ^(bl <<< region ^~~~ ^closed) ^~~~ ^base |||
sr <<< base ^~~~ ^(br <<< closed ^~~~ ^region) ^~~~ ^base |||
ml <<< base ^~~~ ^(ad <<< block ^~~~ ^comps) ^~~~ ^base |||
sr <<< base ^~~~ ^(il <<< region ^~~~ ^closed ^~~~^
region) ^~~~ ^base)
'with' basepairing ... h
region3 = region 'with' (minsize 3)
b)
struct = comps |
singlestrand |
empty
block = singlestrand closed |
comps = block comps |
block |
block singlestrand
singlestrand = region
closed = base region 3 base |
base closed base |
base region closed base |
base closed region base |
base region closed region base |
base block comps base
region3 = base base region
region = base |
base region
base = 'A' | 'C' | 'G' | 'U'

u = f <<< x ^~~~ ^y | | |

g <<< z

says that a tree of type *u *can be built either with operator *f *being applied to subtrees of type *x *and *y*, or with operator *g *being applied to a subtree of type *z*.

Our grammar has been written to exclude structures with isolated base pairs, as such "lonely pairs" can be considered not to occur in native structures. Where such lonely pairs are energetically favourable, this is probably an artefact of the energy model used (Gerhard Steger, personal communication). Optionally, however, our program RNAshapes offers calculations that include such pairs. The grammar also imposes a minimal length of 3 on the turns inside hairpin loops. Readers are invited to derive some trees with this grammar, to assure themselves that invalid structures cannot be derived.

The folding space *F*(*s*) of a sequence *s *is now formally defined as the set of all trees of type struct that exhibit *s *as their sequence of leaves. Care has been taken to ensure that the grammar is non-ambiguous: Each structure can be represented uniquely by a tree, derived by the rules of the grammar in exactly one way. Such non-ambiguity is essential for a mathematically correct probabilistic analysis, as has recently been analysed in [17-19]. Showing the non-ambiguity of a grammar is often difficult. For our grammar, we succeeded with the automatic proof technique presented in [19].

The grammar presented here is actually a simplified version of the full grammar used in our implementation. More details of the full grammar are given in the sections on algorithmics and efficiency analysis.

### Deriving features from structures

As our structures are formulas, we can derive various kinds of information in a most uniform way: We simply interpret the operators as functions operating on a particular domain of interest, such as shapes, energies or strings. Such interpretations consist of one function per operator, and will be called *evaluation algebras*. The value resulting from the interpretation of a structure *x *in algebra *α *will be denoted *x*^*α*^. The convenience of this formalization is that whatever feature of interest we cast in terms of an evaluation algebra, we can be sure that the parsing algorithm that evaluates the folding space can compute this feature [20]. We will specify evaluation algebras that compute free energies, Boltzmann-weighted energies, shapes and string representations of structures. When an interpretation is given, by convention, operator names are converted to lower case and superscripted. This means, for example, that operator *SR *will be given many different interpretations – as function *sr*^*en*^, which adds the free energy increment of stack extension to the enclosed structure's energy, as *sr*^*bw*^, which computes the Boltzmann-weighted energy of the extended substructure in the same situation, as *sr*^*π*^, which computes the stack extension's effect on the shape *x*^*π*^. of structure *x*, and as *sr*^(·)^, which adds another pair of brackets around the string representation of the enclosed substructure.

#### String representation of structures

As a simple first example, we define the interpretation *x*^(·) ^that derives the dot-bracket representation of structure *x*. "(·)" is meant as an abbreviation for "dot-bracket representation".

ss^(·)^(*l*) = _...|*l*| _    (3)

*hl*^(·)^(*a*, *l*, *b*) = (_...|*l*|_)     (4)

*sr*^(·)^(*a*, *x*^(·)^, *b*) = (╫ *x*^(·) ^╫)     (5)

*bl*^(·)^(*a*, *l*, *x*^(·)^, *b*) = (_...|*l*| _╫ *x*^(·) ^╫)     (6)

*br*^(·) ^(*a*, *x*^(·)^, *l*, *b*) = (╫ *x*^(·) ^╫ _...|*l*|_)     (7)

*il*^(·)^(*a*, *l*, *x*^(·)^, *l'*, *b*) = (_...|*l*| _╫ *x*^(·) ^╫ _...|*l'*|_)     (8)

*ml*^(·)^(*a*, *c*^(·)^, *b*) = (╫ *c*^(·) ^╫)     (9)

*ad*^(·)^(*x*^(·)^, *c*^(·) ^= *x*^(·) ^╫ *c*^(·) ^    (10)

*e*^(·) ^= *ε *    (11)

where _...*k *_means *k *dots, |*l*| is the length of string *l*, *ε *denotes the empty string and ╫ is string concatenation. The added characters (,) or . are given in boldface.

#### Free energy

In the energy algebra, each operator, representing a structural feature, is interpreted as a function adding a certain free energy increment to the energy of its embedded substructure(s). Evaluating structure *x *under this interpretation yields energy value *x*^*en*^. Base pairs and stack extensions add stabilising (negative) energies, while most kinds of bulges and loops add destabilising (positive) energies. The concrete energy parameters have been determined experimentally [2,21,22]. We abbreviate them by *δ*_*SR*_, *δ*_*BL *_etc. Using these parameters, we can interpret *SR *by function *sr*^*en *^in the following way:

*sr*^*en *^(*a *, *x*^*en*^, *b) *= *δ *_*SR *_(*a, b*) + *x*^*en *^    (12)

**Figure 1:**
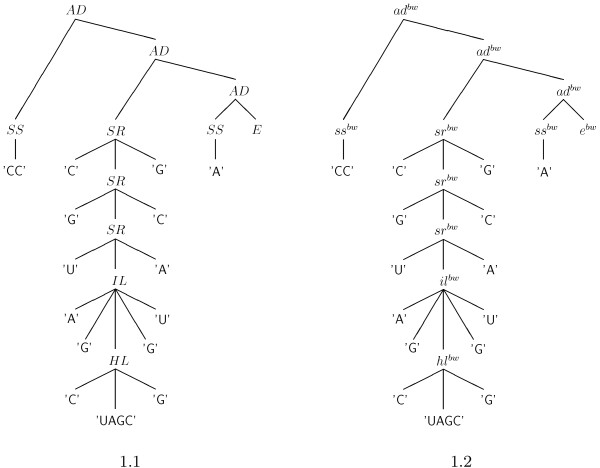
**Different interpretations of operators**. Trees showing different interpretations of operators: 1.1 as symbolic constructors, 1.2 as the tree representation of the formula that computes the Boltzmann-weighted energy of the structure. Note that the trees are isomorphic.

Energy functions for the other operators can be given in a similar way. Comparing *SR*(*a, x, b*) to its free energy interpretation *sr*^*en*^(*a*,*x*^*en*^, *b*), we see that the tree representing a structure has simply been replaced by an isomorphic formula that computes its free energy. (Technically, *a *and *b *have to be base coordinates here, not just bases, since *δ*_*SR *_is defined on stacked base pairs, not just single base pairs.) Under this interpretation, our example structure from Figure 1 becomes

$\begin{array}{ll} ad^{en}(ss^{en}(CC), & ad^{en}(sr^{en}(C,\;sr^{en}(G,\;sr^{en}(U,\;il^{en}(A,\;G,\\ & hl^{en}(C,\;UAGC,\;G),\;G,\;U),\;A),\;C),\;G),\\ & ad^{en}(ss^{en}(A),\;E)))=-4.10kcal/mol \end{array}\;\left(13\right)$

#### Boltzmann-weighted energies

When computing probabilities of structures according to Eq. 2, it is convenient to defer division by *Q *until the very end, and compute Boltzmann-weighted energies instead, according to the equation *x*^*bw *^= $e^{-x^{en}/RT}$

Hence, the Boltzmann-weighted energy algebra can be derived from the free energy algebra:

$\begin{array}{ccc} sr^{bw}(a,x^{bw},b) & = & e^{-sr^{en}(a,x^{en},b)/RT}\;\left(14\right)\\ & = & e^{-(\delta_{SR}(a,b)+x^{en})/RT}\;\left(15\right)\\ & = & e^{-\delta_{SR}(a,b)/RT}\cdot e^{-x^{en}/RT}\;\left(16\right)\\ & = & e^{-\delta_{SR}(a,b)/RT}\cdot x^{bw}\;\left(17\right) \end{array}$

Again, the other functions can be derived in a similar way. Like energies summate over substructures; Boltzmann-weighted energies multiply.

For our example structure, the Boltzmann-weighted energy is 774.6261 and *Q = *793.9457, resulting in a probability *P *= 0.9756663.

#### Shapes

RNA abstract shapes are a generic concept. They are defined by means of abstraction functions preserving varying amounts of detail. These functions are homomorphisms from structures to another tree-like domain, preserving adjacency and nesting of substructures. For the trees representing shapes, we use 4 operators: *OP *("open") represents the shape of all structures without base pairs, *CL *("closed") represents a helical region, *AD *and *E *are re-used here to represent lists of adjacent (sub)shapes. We present two shape abstraction functions *π*5 and *π*3, known as level-5 and level-3 abstraction [16].

$\begin{array}{llllll} ss^{\pi 5}(l) & = & OP & ss^{\pi 3}(l) & = & OP\\ hl^{\pi 5}(a,l,b) & = & CL(E) & hl^{\pi 3}(a,l,b) & = & CL(E)\\ sr^{\pi 5}(a,x^{\pi 5},b) & = & x^{\pi 5} & sr^{\pi 3}(a,x^{\pi 3},b) & = & x^{\pi 3}\\ bl^{\pi 5}(a,l,x^{\pi 5},b) & = & x^{\pi 5} & bl^{\pi 3}(a,l,x^{\pi 3},b) & = & CL(x^{\pi 3})\\ br^{\pi 5}(a,x^{\pi 5},l,b) & = & x^{\pi 5} & br^{\pi 3}(a,x^{\pi 3},l,b) & = & CL(x^{\pi 3})\\ il^{\pi 5}(a,l,x^{\pi 5},l^{\prime },b) & = & x^{\pi 5} & il^{\pi 3}(a,l,x^{\pi 3},l^{\prime },b) & = & CL(x^{\pi 3})\\ ml^{\pi 5}(a,x^{\pi 5},b) & = & CL(x^{\pi 5}) & ml^{\pi 3}(a,x^{\pi 3},b) & = & CL(x^{\pi 3})\\ ad^{\pi 5}(x^{\pi 5},y^{\pi 5}) & = & \text{if }x^{\pi 5}=OP & ad^{\pi 3}(x^{\pi 5},y^{\pi 3}) & = & \text{if }x^{\pi 3}=OP\\ & & \text{then }y^{\pi 5} & & & \text{then }y^{\pi 3}\\ & & \text{else }AD(x^{\pi 5},y^{\pi 5}) & & & \text{else }AD(x^{\pi 3},y^{\pi 3})\\ e^{\pi 5} & = & E & e^{\pi 3} & = & E \end{array}$

**Figure 2:**
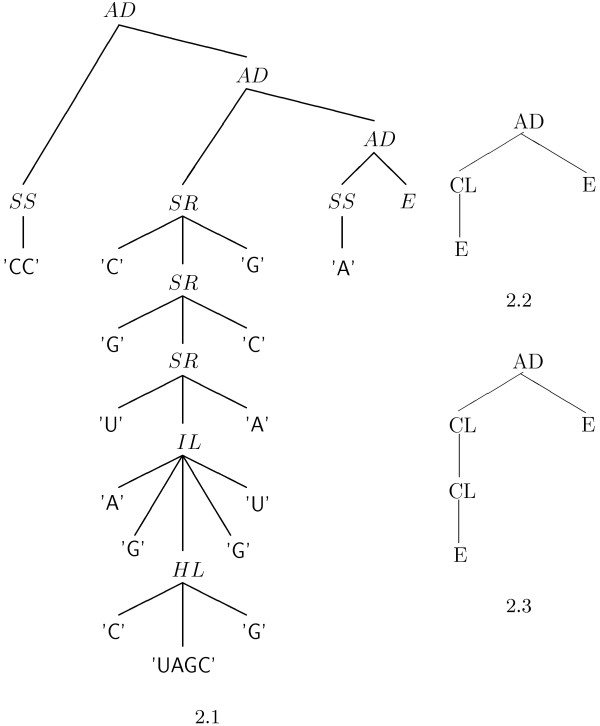
**Structures and shapes**. Trees representing 2.1 a structure and its shapes according to 2.2 π5 and 2.3 π3.

*π*5 maps all helices to the *CL *operator, abstracting from helix length and interruptions by bulges or internal loops. Except for the completely unpaired structure, no single-stranded regions at all are retained. In contrast to this, *π*3 introduces a new *CL *operator in the shape tree, whenever a helix is interrupted by a bulge or internal loop. However, the type of interruption is not recorded. Figure 2 shows a structure and the two shape trees according to *π*5 and *π*3.

### Complete probabilistic shape analysis

Given the tree grammar defining valid structures, and a particular RNA sequence *s*, a parsing algorithm can construct the complete folding space *F*(*s*). For all *x *∈ *F*(*S*), it can compute values *x*^*en*^, *x*^*bw*^, * x*^*π *^and *x*^(·)^, where *π *is one of our shape abstraction functions. We shall make use of the notion of an *exploded folding space*, which is a list of derived values, one for each member of *F*(*s*). Exploded folding spaces are convenient for formal definitions, but they must be avoided in actual computations, as their size increases exponentially with the length of *s*. This is possible with dynamic programming.

To explain the model underlying complete probabilistic shape analysis, we proceed in three steps. We first review simple shape analysis (as described in [16]). Then we describe the computation of the partition function as well as the structure of maximal Boltzmann weight. Finally, we combine the objectives of both types of analysis to define shape probabilities.

#### Simple shape analysis

Consider the list *L*_*shreps *_of all shape-representative structures (*shreps*) for *s*, together with their energies and shapes, and sorted on the energy component. Here, *π *(*F*(*s*)) is short for {*x*^*π*^*x *∈ *F*(*s*)}; it is called the shape space of *s*.

*L*_*shreps*_(*s*) = [(*x*^(·)^, *x*^*en*^, *x*^*π*^)|*x*^*π *^∈ *π*(*F*(*s*)), *x*^*en *^= *min*{*z*^*en*^|*z *∈ *F*(*s*), *z*^*π *^= *x*^*π*^}]     (18)

For given *k*, simple shape analysis computes the first *k *elements of this list, in *O*(*kn*^3^) time and *O*(*n*^2^) space.

#### Computing Boltzmann-weighted energies

Our new goal is to compute Boltzmann-weighted energies of shapes, defined as the accumulated Boltzman-weighted energies of all structures within a shape. We want to compute the value triple

$B=(Q,x_{opt}^{bw},x_{opt}^{\left(\cdot \right)})\;\left(19\right)$

where $Q=\sum \left\{x^{bw}|x\in F(s)\right\}$, the sum of all Boltzmann-weighted energies, and *x*_*opt *_∈ *F*(*s*) is the structure of maximal Boltzmann-weighted energy.

We define a function *h*_*B*_, which computes *B *from the exploded folding space

*L*_*bw*_(*s*) = [(*x*^*bw*^, *x*^*bw*^, *x*^(·)^) | *x *∈ *F*(*s*)].     (20)

by accumulation of Boltzmann-weighted energies in the first component and maximization of Boltzmann-weighted energies in the second, while recording the structure of highest Boltzmann weight in its string representation in the third component. For a single structure, both energy sum and individual energy coincide, which explains why *x*^*bw *^occurs twice in Equation 20. Details are given in the Methods section.

#### Computing shape probabilities

Our ultimate goal is to compute the probabilities of all shapes in *π*(*F*(*s*)). To this end, we need to accumulate Boltzmann-weighted energies *per shape*. The accumulated weight of shape *p *is

$x_{\sum p}^{bw}=\sum \left\{x^{bw}|x\in F(s),x^{\pi }=p\right\}\;\left(21\right)$

and the shape's probability is $x_{\sum p}^{bw}/Q$.

Along with the accumulated weights, we also want to compute the shapes themselves, together with their shreps $x_{opt,p}^{\left(\cdot \right)}$ and their Boltzmann-weighted energies $x_{opt,p}^{bw}$. (The latter will eventually be converted back to free energies in the output of our program.) Our desired result is therefore a complete list of all these values, in the form

$P=[(p,(x_{\sum p}^{bw},x_{opt,p}^{bw},x_{opt,p}^{\left(\cdot \right)}))|p\in \pi (F(s))]\;\left(22\right)$

We define a function *h*_*P *_that computes P from the exploded folding space

*L*_*sh *_(*s*) = [(*x*^*π*^, (*x*^*bw*^, *x*^*bw*^, *x*^(·)^))|*x *∈ *F*(*s*)]     (23)

by computing shape abstractions in the first component, and applying *h*_*B *_on the other components in a shape-wise fashion. Details are given in the Methods section.

We now report our findings from applications of complete probabilistic shape analysis to various types of RNA.

### Transfer RNA

**Figure 3:**
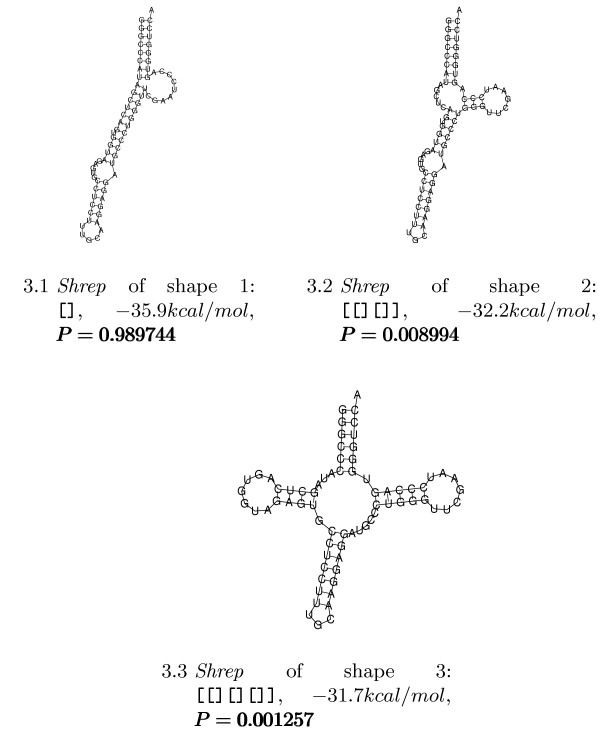
**Shreps of the *N. pharaonis *tRNA-ala**. *Shreps *of the three most probable shapes of the *N. pharaonis *tRNA-ala together with the probabilities of the shapes (sorted by increasing energy).

Applying the shape probability algorithm to the alanine tRNA of *Natronobacterium pharaonis *(embl:AB003409.1) gives the results shown in Figure 3.

The shape holding the MFE-structure (shape 1) shows a high probability, whereas the other shapes have probabilities below 1%. This means that this unmodified RNA is very unlikely to occur in the cloverleaf shape. This is consistent with biological knowledge and clearly expresses the need for other mechanisms, such as base modifications, to ensure that the cloverleaf structure is actually achieved.

### Attenuator

**Figure 4:**
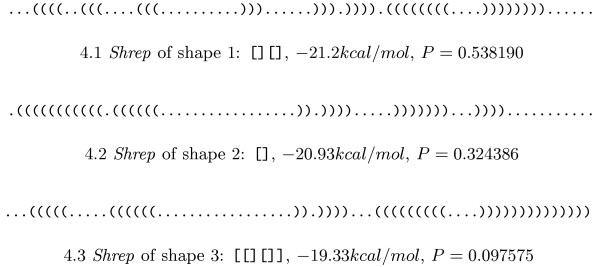
**Shreps of the Attenuator**. *Shreps *of the three most probable shapes of the Attenuator together with the probabilities of the shapes (sorted by increasing energy). Together, they cover 0.95 probability, ruling out further shapes of biological importance.

The pheS-pheT-Attenuator of *E. coli *(embl:V00291.1/3682-3746) is known to switch from a translationally inactive to a translationally active conformation under specific conditions. These two conformations correspond to two valleys in the structure landscape that are separated by a saddle point (energy barrier). In terms of shape analysis, this means that two shapes should be present with reasonable probability. The corresponding experiment yields the results summarised in Figure 4. The analysis shows that Shapes 1 and 3 are rather similar with respect to their *shreps*, so their probabilities can be added. This means that the shape with two hairpins, which may be embedded in a multiloop, has a probability of 0.635765 and the shape with one hairpin has a probability of 0.324386. Shape 1+3 corresponds to the "off" position of the switch and the higher probability inidcates that this is its native position. The "on" position (Shape 2) is less probable, indicating the need for some external effector to trigger the switch.

### Leader of ptsGHI

**Figure 5:**
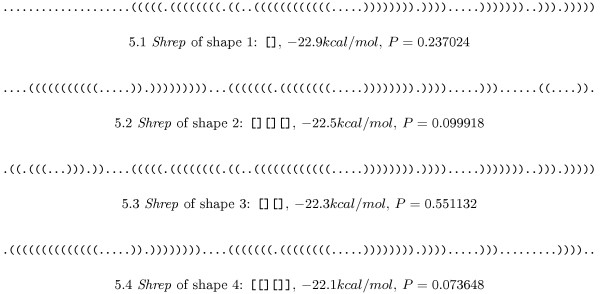
**Shreps of the leader of the *ptsGHI *operon**. *Shreps *of the four most probable shapes of the leader of the *ptsGHI *operon in *B. subtilis *together with the probabilities of the shapes (sorted by increasing energy).

The ptsGHI operon in *B. subtilis *(gb:Y11193/1016-1107) includes the genes involved in glucose transport by the phosphotransferase system. In [23], it was shown that expression of this operon is controlled at the level of transcript elongation by a protein-dependent riboswitch. In the absence of glucose, a transcriptional terminator prevents elongation into the structural genes. In the presence of glucose, the GlcT protein is activated and binds and stabilises an alternative structure that overlaps the terminator and prevents termination. Applying RNAshapes to calculate probabilities of shapes gave the results shown in Figure 5. The first striking result of this analysis is that a shape holding an energetically less favourable *shrep *(shape 3) is the most probable one. Again, we can further merge shapes by looking at their *shreps*. Only *Shreps *2 and 4 carry the antiterminator hairpin (the small hairpin at the 5'-end of *shrep *3 does not alter the terminator hairpin), whereas *Shreps *1 and 3 carry the terminator hairpin. Summation of the probabilities gives 0.788176 for the terminating conformations and 0.173566 for the read-through conformations. This corresponds to experimental results showing that the switch is natively in the "off" position and is triggered by the GlcT protein to enable transcript elongation.

### Precursor of microRNA lin-4

microRNAs (miRNAs) are small (~22 nt) regulatory RNAs that are processed from larger precursors, for which the secondary structure is assumed to play an important role. A common feature of all known precursors is that they form a hairpin with significantly lower energy than for random sequences of the same dinucleotide distribution [24]. This suggests a well-defined secondary structure, which implies that the corresponding shape should have a very high probability. An analysis of the precursor of *C. elegans *lin-4 (miRBase:MI0000002) [25] reveals the shape [] with probability 0.999996, which means that only 1 in 250,000 molecules has a different shape, or that each molecule spends 99.9996% of its lifetime in the single hairpin shape. A probability cut-off of 10^-6 ^was used for the output, which might be the reason that no further shapes appear.

**Figure 6:**
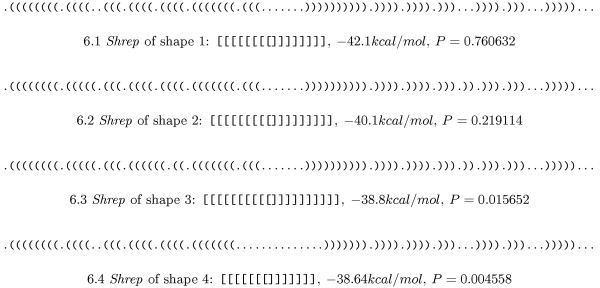
**Shreps of the four most probable shapes of the *C. elegans *lin-4 precursor**. *Shreps *of the four most probable shapes of the *C. elegans *lin-4 precursor at shape abstraction level 3, together with the shape probabilities (sorted by decreasing probability).

Another fact that has to be considered is that the shape abstraction might have been too strong. For this reason, we performed an analysis with abstraction level 3, retaining more structural detail, and giving the results shown in Figure 6.

All *shreps *are very similar, so it is reasonable to combine them in the single hairpin shape. Their probability sum is 0.999956, which except for rounding inaccuracies is the same as the probability of the single hairpin shape.

### mRNA

**Figure 7:**
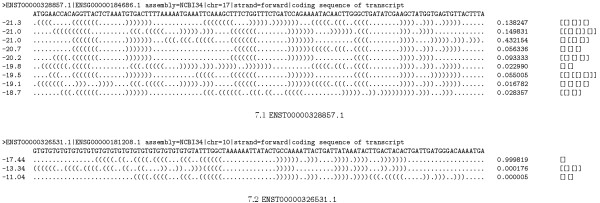
**Shape probabilities of non-structural and structural RNAs**. The sequence in 7.1 shows a result which one would expect for coding sequences where structure plays no role, while 7.2 shows a coding sequence that seems to have a rather well-defined structure.

The previous sections show how the probabilities of shapes can be used to analyse functional RNAs and their specific structural properties. But what about messenger RNA (mRNA)? As the structure of an mRNA is generally assumed to be less important, and because evolution has to ensure the correct coding of amino acids, we would expect the results to be inclonclusive. Interestingly, analyses of numerous mRNA coding sequences revealed a wide variety of results. In Figure 7 we give two examples that can be seen as extremal observations. The "expected" case is observed for ENST00000328857.1 (see Figure FIG:CDS:PROBS:A), where we find nine shapes with remarkably high probabilities, and four cases of "overtaking", where the shape probability ranking contradicts the ranking of shreps by energy. The other extreme is observed for ENST00000326531.1 (see Figure FIG:CDS:PROBS:B), where we find a hairpin shape with probability 0.999819 inside the coding sequence. Reasons for this diversity of results may be that at least some coding sequences carry structural features necessary for correct function, or that structure, no matter whether well-defined or ill-defined, was never selected for or against.

### Approximating shape probabilities via sampling

In the Methods section, we show that complete probabilistic shape analysis has a "slow" exponential term in its runtime requirements. This makes probabilistic shape analysis unfeasible for long sequences. Hence, to allow the analysis of such sequences, we combine the stochastic sampling introduced in [4] with a-posteriori shape abstraction. A sample from the structure space holds M structures together with their shapes, on which classification is performed. The probability of shape *p *can then be approximated by its frequency *f*_*p *_in the sample. The accuracy of this approach depends on the sample size *M*, which must be large enough to achieve statistical confidence. Assuming the counts to have a Poisson distribution with parameter *Mp*, we can approximate them with a normal distribution with mean *Mp *and variance *Mp *for large sample sizes. To allow 10% deviation of the estimated frequency *f*_*p *_within a 95% confidence interval, we require that $2\sqrt{\mathrm{var}}$ ≤ 0.1 mean or *Mp *≥ 400, or, with lspoi being the lowest shape probability of interest, *M *≥ 400/lspoi. Thus, *M *= 1000 is sufficient to achieve reasonable results for the shapes with high probability, and this number is independent of the sequence length. This result was confirmed by empirical analyses (see Table 3).

**Table 3:** Comparison of sampling frequencies and exact probabilities. Comparison of sampling frequency and exact probability for the four most probable shapes of the pheS-pheT-Attenuator from *E. coli*; the Spliced Leader of *L. collosoma *(gb:S76723/1-56) and the leader of the HIV-1 genome (gb:K02013/1-281), all of which are conformational switches. The sample size for each was 1000 and the analyses were repeated 1000 times.

Shape	Frequency	Probability
pheS-pheT-Attenuator (74nt)		

[] []	0.538146 ± 0.012546	0.5381897
[]	0.324908 ± 0.011745	0.3243859
[[] []]	0.097263 ± 0.007509	0.0975747
[] [] []	0.038984 ± 0.004872	0.0388670

Spliced Leader (56nt)		

[[[[[]]]]]	0.4966 ± 0.012635	0.4962782
[[[[]]]]	0.348569 ± 0.011618	0.3491818
[[[]]]	0.060008 ± 0.005976	0.0595903
[[]]	0.056138 ± 0.005741	0.0559218

HIV-1 Leader (281nt)		

[] [[] [[] []]]	0.629139 ± 0.015878	0.6164011
[] [[[] [[] []]] []]	0.337976 ± 0.014817	0.3492262
[[] [] [[[] [[] []]] []]]	0.017246 ± 0.003252	0.0169983

The remaining question is, when to use the exact algorithm and when the sampling? The running times for the two methods are summarised in Table 4 and show that for sequences up to 100 nt the exact algorithm should be used, whereas for longer sequences the sampling algorithm is favourable.

**Table 4:** Comparison of running times for the exact algorithm and the sampling approach. Comparison of running times for the exact algorithm and the sampling approach (1000 samples) on an Intel Xeon 2.8 GHz CPU.(*n *= sequence length; * computed on an UltraSparc III 900 MHz using 64-bit.)

*n*	Sampling	Exact Algorithm
57 nt	6.42 s	0.33 s
74 nt	17.36 s	0.93 s
94 nt	69.56 s	31.85 s
108 nt	36.24 s	57.43 s
130* nt	184.85 s	12016.68 s

More recently, the approach of [4] was extended by a clustering step and computation of centroids for each cluster [26]. The clusters can be seen as analogous to our shapes, though without an explicit notion of abstraction.

## Discussion

### Mathematical and algorithmic aspects

Abstract shapes are a mathematically precise, intuitively simple and non-heuristic means for partitioning the folding space into classes of structures. They enable us to derive synoptic properties of these classes such as the *shreps *of each class [16] or the accumulated class probabilities, as introduced here. The granularity of the partitioning can be adapted to the length of the sequence by choosing different abstraction levels. Simple shape analysis is possible with the same algorithmic complexity as standard MFE folding (*O*(*n*^3^)), while probabilistic shape analysis requires *O*(*n*^3^·*P*^*n*^), where P depends on the abstraction level chosen (see Methods). When the exponential term becomes problematic, one can switch to probabilistic sampling with subsequent shape classification. However, there remains the challenge of finding an algorithm for probabilistic analysis for the *k *best shapes that avoids the *O*(*P*^*n*^) factor.

Given such satisfactory mathematical and algorithmic properties, the question of whether shapes constitute an abstraction that is also biologically meaningful must not be overlooked.

### Biological adequacy of shapes

Our idea is that a shape class comprises similar structures that can potentially perform the same function – with the consequence that looking at the *shreps *and their shape probabilities gives us a precise and complete account of a molecule's functional potential. This incurs the risk of overlooking an important feature when shapes exhibit substantial internal variation, while only their *shrep *is submitted for further scrutiny.

**Figure 8:**
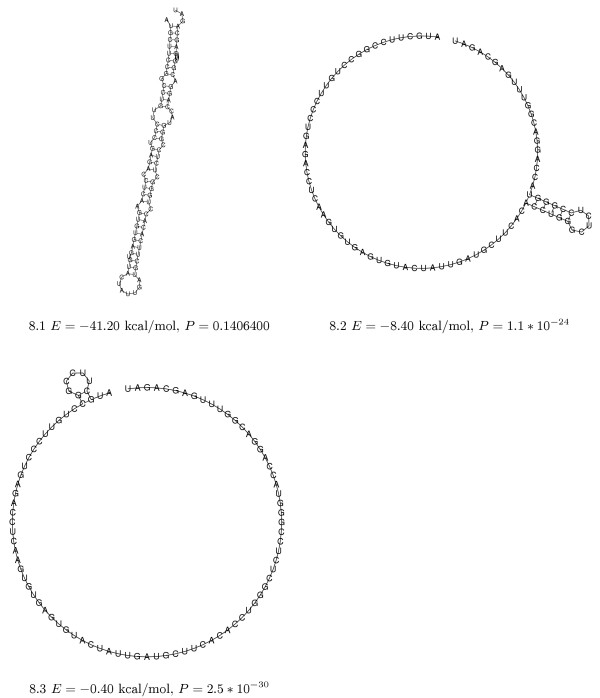
**Variation within shape**. Three members of the [] shape of C. elegans lin-4 miRNA precursor. The structure shown in 8.1 is the shrep of the [] shape and also the MFE-structure. 8.2 and 8.3 show members which are structurally dissimilar to the shrep. Note the very low probabilities of the latter two.

We address this concern about variation within shape by two examples. In Figure 8, we show three extremal members of shape [] for the *C. elegans *lin-4 miRNA precursor.

**Figure 9:**
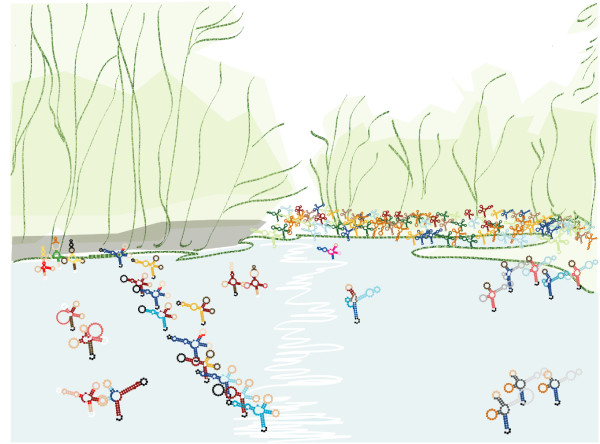
**tRNA cloverleaf shape members (skating on a winter pond)**. Complete snapshot of 127 low-energy members of the cloverleaf shape of *Myc. capricolum *tRNA-Leu in the energy range of 6 kcal/mol above the MFE. All resemble the *shrep *very closely. Artistic arrangement by S. Konermann.

This example shows that a shape can (and will) hold structures with little similarity to that of the *shrep*. But note that these structures are taken from energy ranges high above the MFE, and therefore should not play a functional role. Our second example shows 116 members of the cloverleaf shape of *Myc. capricolum *tRNA-Leu (embl:X16754.1) (Figure 9). This is a complete snapshot of the low-energy membership of shape [[] [] []] in the range of 3.1 kcal/mol above the MFE. All the members in this energy range resemble the *shrep*.

From our observations so far, it appears that the low-energy segment shows low variation within shape. Nevertheless, a deeper mathematical analysis or empirical study of variation within shape is desirable.

### Significance of predicted structures

There has been an ongoing debate about the significance of low free energies achieved by a structure [24,27-30]. It is significant because of the need to assign some significance measure to a structure prediction. In various ways, these approaches compare the MFE of the native sequence to that of random sequences of the same mono- or dinucleotide distribution. According to the most recent study [31], several classes of structural RNA, such as type III hammerhead ribozymes and U2 small nucleolar RNAs, have lower folding energy than random RNA of the same dinucleotide frequency.

## Conclusion

Probabilistic shape analysis allows us to approach the question of the significance of predicted structures from a new angle. We take the view that in the evolution of structure, competition comes not so much from sequence variation (akin to randomizing effects) as from alternative structures within the folding space. A different structure can come to dominate the native one because of very few mutational events, with the effect that a functional RNA becomes nonfunctional. Hence, evolution should strive to make the shape of a functional structure clearly dominate over other shapes. This not only stabilizes this structure in the overall Boltzmann ensemble of the native sequence, but also protects against immediate loss of function because of a small number of mutations, so that there is time (on an evolutionary scale) to restabilize the structure by compensatory mutations, which we often observe.

This line of thought is consistent with our observations reported here, but it is by no means proven. An observation like that for ENST00000326531.1 calls for further statistical or experimental work, to disprove or prove the biological relevance of a structural motif with shape probability 0.999819 inside this coding sequence. We propose that dominance of shape should be further investigated as a measure of structural significance.

## Methods

### Algorithmics and efficiency analysis

In this section we are concerned with two algorithmic problems:

1. We define our objective functions *h*_*B *_and *h*_*P *_and show that they can be implemented via dynamic programming.

2. We analyse and discuss the asymptotic efficiency of complete probabilistic shape analysis achieved by this implementation.

We shall define our objective functions *h*_*B *_and *h*_*P *_as evaluators of an exploded folding space.

Subsequently, we will show that they can be computed efficiently via dynamic programming because they satisfy the condition known as Bellman's Principle. Thus, we start with a discussion of this principle.

### Notation

We define objective functions on lists. The empty list is denoted as []. [*x*_1_, ..., *x*_*n*_] is the list of *n *elements *x*_1 _to *x*_*n *_where *n *can also be zero, the list thus being empty. In expressions like [...|*x*_1 _← *z*_1_,...,*x*_*n *_← *z*_*n*_] the leftarrow ← denotes list membership. The operator ╫ concatenates two lists. The function map applies a function elementwise to a list. The function concat concatenates a list of lists into a single list. As above, features that can be derived from a structure *x *are its free energy *x*^*en*^, its Boltzmann weight *x*^*bw *^and its notation as a "Vienna string" *x*^(·)^. According to their subscripts, Boltzmann weights can be summed-up ($x_{\sum }^{bw}$) or optimised ($x_{opt}^{bw}$). $x_{opt}^{\left(\cdot \right)}$ is the Vienna string of an energetically optimal (sub-)structure *x*. The shape of structure *x *is denoted by *x*^*π*^.

### Bellman's Principle

Bellman's Principle [32] captures the essence of dynamic programming. It states the conditions under which a search space can be pruned by applying an objective function at each intermediate step, whenever a list of alternative intermediate results has been obtained. Solutions to sub-optimal subproblems can be discarded, yet the overall optimal solution will be found.

Bellman's Principle can be formally captured in the following two equations (cf. [20]):

*h*([*f*(*x*_1_,...,*x*_*k*_)) | *x*_1 _← *z*_1_,...,*x*_*k *_← *z*_*k*_]) = *h *([(*f*(*x*_1_,...,*x*_*k*_)) | *x *← *h *(*z*_1_),...,*x*_*k *_← *h*(*z*_*k*_)])     (24)

*h*(*z*_1 _╫ *z*_2_) = *h*(*h*(*z*_1_) ╫ *h*(*z*_2_))     (25)

where the *z*_*i *_denote lists of intermediate results, ← denotes list membership, and ╫ denotes list concatenation. In our context, functions *f *will be *sr*^*en*^, *sr*^*bw *^and so on.

It is not generally true that a function that computes the desired result from the exploded search space will also produce this result when applied at intermediate steps in a dynamic programming algorithm. But it is easy to see that the conditions 24 and 25 together guarantee that this is in fact the case.

### Definition and justification of objective function *h*_*B*_

Function *h*_*b *_computes *B *= ($x_{\sum }^{bw}$, $x_{opt}^{bw}$, $x_{opt}^{\left(\cdot \right)}$) from the exploded folding space

*L*_*bw*_(*s*) = [(*x*^*bw*^*,x*^*bw*^*,x*^(·)^)|*x *∈ *F*(*s*)] (see Equations 19 and 20).

#### Definition of *h*_*B*_

$\begin{array}{ccc} h_{B}([s_{1},\ldots ,s_{n}]) & = & h^{\prime }([],\;[s_{1},\ldots ,s_{n}]),\text{ where}\\ h^{\prime }(p,[]) & = & p\\ h^{\prime }(p,[s_{1},\ldots ,s_{n}]) & = & h^{\prime }(\text{insert}(s_{1},p),[s_{2},\ldots ,s_{n}])\\ \text{insert((}x_{\sum }^{bw},x_{opt}^{bw},x^{\left(\cdot \right)}),[]) & = & \left[(x_{\sum }^{bw},x_{opt}^{bw},x^{\left(\cdot \right)})\right]\\ \text{insert}((x_{\sum }^{bw},x_{opt}^{bw},x^{\left(\cdot \right)}),[(y_{\sum }^{bw},y_{opt}^{bw},y^{\left(\cdot \right)})]) & = & \{\begin{array}{cc} {}[(y_{\sum }^{bw}+x_{\sum }^{bw},y_{opt}^{bw},y^{\left(\cdot \right)})], & \text{if }y_{opt}^{bw}\geq x_{opt}^{bw}\\ {}[(y_{\sum }^{bw}+x_{\sum }^{bw},x_{opt}^{bw},x^{\left(\cdot \right)})], & \text{if }y_{opt}^{bw}<x_{opt}^{bw} \end{array} \end{array}\;\left(26\right)$

where *s*_*i *_= ($x_{i}^{bw}$, $x_{i}^{bw}$, $x_{i}^{\left(\cdot \right)}$), $x_{\sum }^{bw}$ is a sum of Boltzmann-weighted energies, $x_{opt}^{bw}$ is the optimal Boltzmann-weighted energy seen so far, and *x*^(·) ^is the string representation of a structure with this Boltzmann-weighted energy; likewise for *y*. Thus, this choice function sums up Boltzmann-weighted energies of structures and keeps track of the structure that has the optimal energy. The result list has at most one element.

The function *h *not only extracts the desired information from the exploded folding space, it also satisfies Bellman's Principle of Optimality, as will be shown next.

#### Justification of objective function *h*_*B*_

Equation 25 is easy to verify: every $x_{\sum }^{bw}$ is added once and the optimal structure is determined by looking at each one on the left-hand side once and on the right-hand side twice, which gives the same result. For Equation 24, we have to differentiate between three kinds of operators: the first is of the form *f *= *c*, meaning it is a constant that does not depend on sub-solutions (operators *SS*, *HL *and *E*); the second is of the form *f *= *a·x*, where a scalar *a *is multiplied with an evaluated sub-solution *x *(operators *SR*, *BL*, *BR*, *IL*, and *ML*); the third is of the form *f *= *a*·*x*_1_·*x*_2_, where a scalar *a *and the values of two sub-solutions are multiplied (operator *AD*). The first case (*f *= *c*) is trivial, since we do not have any answer lists *z*, and *h *is thus applied only once. In the second case ( *f *= *a*·*x*), we sum up terms using *a*·*x*_1 _+ *a*·*x*_2_, which is equal to *a*·( *x*_1 _+ *x*_2_), or choose the maximum using max ( *a*·*x*_1 _, *a*·*x*_2_) which is equal to *a*·max ( *x*_1_, *x*_2_). In the third case ( *f *= *a*·*x*_1_·*x*_2_), we sum up terms using $a\cdot x_{1}^{1}\cdot x_{1}^{2}+a\cdot x_{1}^{1}\cdot x_{2}^{2}+a\cdot x_{2}^{1}\cdot x_{1}^{2}+a\cdot x_{2}^{1}\cdot x_{2}^{2}$), which is equal to *a*·($x_{1}^{1}+x_{2}^{1}$)·($x_{1}^{2}+x_{2}^{2}$), or choose the maximum using max ($a\cdot x_{1}^{1}\cdot x_{1}^{2},a\cdot x_{1}^{1}\cdot x_{2}^{2},a\cdot x_{2}^{1}\cdot x_{1}^{2},a\cdot x_{2}^{1}\cdot x_{2}^{2}$), which is equal to *a*·max ($x_{1}^{1},x_{2}^{1}$)·max ($x_{1}^{2},x_{2}^{2}$).

### Definition and justification of objective function *h*_*P*_

Function *h*_*P *_computes *P *= [(*p*, ($x_{\sum p}^{bw}$, $x_{opt,p}^{bw}$, $x_{opt,p}^{\left(\cdot \right)}$))| *p *∈ *π *(*F*(*s*))] from the exploded folding space *L*_*sh*_(*s*) = [(*x*^*π*^, (*x*^*bw*^,*x*^*bw*^, *x*^(·)^)) |*x *∈ (*F*(*s*)) ](See Equations 22 and 23).

#### Definition of objective function *h*_*P*_

Using the previous objective function *h*_*B*_, we define the classifying objective function *h*_*P *_as:

*h*_*P*_(*z*) = concat (map(*h*_*B*_, split(*z*)))     (27)

where map applies function *h*_*B *_elementwise to a list,

map (*f*, [*a*_1_,..., *a*_*r*_]) = [*f*(*a*_1_),..., *f*(*a*_*r*_)]     (28)

and concat concatenates a list of lists into a single list.

Function split splits answer list *z *into sublists, one for each occuring shape:

$\begin{array}{l} \text{split}(z)=\text{map}(\text{reverse},{\mathrm{split}}^{\prime }([],z))\\ {\mathrm{split}}^{\prime }(cs,[])=cs\\ {\mathrm{split}}^{\prime }(cs,[s_{1},\ldots ,s_{n}])={\mathrm{split}}^{\prime }(\text{insert}(s_{1},cs),[s_{2},\ldots ,s_{n}])\\ \text{insert}((x_{s},x),[])=[(x_{s},x)]\\ \text{insert}((x_{s},x),[{\left(y_{s},y\right)}_{1},\ldots ,{\left(y_{s},y\right)}_{m}])\\ =\{\begin{array}{ll} {}[{\left(y_{s},x:y\right)}_{1},\ldots ], & \text{if }x_{s}=y_{s}\\ {}[{\left(y_{s},y\right)}_{1},\text{insert}((x_{s},x),[{\left(y_{s},y\right)}_{2},\ldots ])], & \text{if }x_{s}\neq y_{s} \end{array}\;\left(29\right) \end{array}$

where *cs *is a list of classes (shape-attributed lists of data), *x*_*s *_and *y*_*s *_are attributes (shapes), and *x *and *y *are the data to be classified. (*x*:*y*) inserts *x *into the front of list *y*. reverse reverses a list. ( , )_*m *_denotes the *m*-th (last) element of a list of pairs.

This definition of the classifying objective function can be rewritten as:

$\begin{array}{l} \hat{h}([s_{1},\ldots ,s_{n}])={\hat{h}}^{\prime }([],[s_{1},\ldots ,s_{n}])\\ {\hat{h}}^{\prime }([p_{1},\ldots ,p_{m}],[])=[p_{1},\ldots ,p_{m}]\\ {\hat{h}}^{\prime }([p_{1},\ldots ,p_{m}],[s_{1},\ldots ,s_{n}])={\hat{h}}^{\prime }(\text{insert}(s_{1},[p_{1},\ldots ,p_{m}]),[s_{2},\ldots ,s_{n}])\;\left(30\right)\\ \text{insert}((x_{s},(x_{\sum }^{bw},x_{opt}^{bw},x_{opt}^{\left(\cdot \right)})),[])=[(x_{s},(x_{\sum }^{bw},x_{opt}^{bw},x_{opt}^{\left(\cdot \right)}))]\\ \text{insert}((x_{s},(x_{\sum }^{bw},x_{opt}^{bw},x_{opt}^{\left(\cdot \right)})),[\;{\left(y_{s},(y_{\sum }^{bw},y_{opt}^{bw},y_{opt}^{\left(\cdot \right)})\right)}_{1},\\ {\left(y_{s},(y_{\sum }^{bw},y_{opt}^{bw},y_{opt}^{\left(\cdot \right)})\right)}_{2},\ldots ,{\left(,\right)}_{m}])\\ =\{\begin{array}{ll} {}[(y_{s,1},(y_{\sum ,1}^{bw}+x_{\sum }^{bw},y_{opt,1}^{bw},y_{opt,1}^{\left(\cdot \right)})),\ldots ], & \text{if }y_{s,1}=x_{s}\text{ and }y_{opt,1}^{bw}\geq x_{opt}^{bw}\\ {}[(y_{s,1},(y_{\sum ,1}^{bw}+x_{\sum }^{bw},x_{opt}^{bw},x_{opt}^{\left(\cdot \right)})),\ldots ], & \text{if }y_{s,1}=x_{s}\text{ and }y_{opt}^{bw}<x_{opt}^{bw}\\ {\left(y_{s},(y_{\sum }^{bw},y_{opt}^{bw},y_{opt}^{\left(\cdot \right)})\right)}_{1}: & \\ \text{insert}((x_{s},(x_{\sum }^{bw},x_{opt}^{bw},x_{v})), & \\ {}[{\left(y_{s},(y_{\sum }^{bw},y_{opt}^{bw},y_{v})\right)}_{2},\ldots ,{\left(,\right)}_{m}]), & \text{if }y_{s,1}\neq x_{s} \end{array} \end{array}$

where ( , )_*m *_again denotes the *m*-th (last) element of a list of pairs. Note that list [*p*_1_,...,*p*_*m*_] can be empty. For long lists of classes, the above insert function is inefficient, since on average it has to run through half the list elements. This can be remedied by using a more efficient data structure, e.g. a hash of which the keys are shapes.

Given this definition, it is clear that $\hat{h}$ computes the desired information from the exploded folding space *L*_*sh*_. That this can also be achieved efficiently via dynamic programming needs yet to be established.

### Dynamic programming with classification

By *dynamic programming with classification *we refer to an analysis that splits up the search space into (disjoint) classes, and applies some objective function class-wise. The classes are not predefined, but arise from information that is also derived from the search space. Almost certainly, this type of problem has arisen before in the 50 year history of dynamic programming. To the best of our knowledge, however, it has not been described as a generic method.

In DP with classification, we compute a classification attribute together with each score value, such that scores can be attributed to a particular class of (sub)solutions. The classification attribute is derived by interpreting solutions in a particular algebra, in our context the shape algebra. We then apply the optimization objective *h *separately for each class, returning, for example, the *k *best scores for each solution class. To this end, we must provide a classification function ${\hat{f}}_{i}$ for each *f*_*i*_, such that we can compute (attribute/score) pairs: (${\hat{f}}_{i}$ ×*f*_*i*_) ($\hat{a}$, *a*), ($\hat{b}$,*b*)) = ($\hat{f}$ ($\hat{a}$, $\hat{b}$), *f *(*a*, *b*)). The function $\hat{h}$ applies *h *separately on each class and is defined (consistent with our earlier definition) as

$\hat{h}$(*x*) = concat (map ( *h*, split (*x*)))     (31)

where split separates a list of attribute/value pairs into sublists, one for each attribute occurring, (map (*h, z*)) applies *h *to each sublist of *z*, and concat rejoins sublists.

We show that $\hat{h}$ satisfies Bellman's Principle when *h *does. This means that classification can be applied with any DP algorithm.

#### Theorem

When *h *satisfies Bellman's Principle, and $\hat{h}$ is defined as above, then $\hat{h}$ also satisfies Bellman's Principle, i.e. we have for each list *xs, ys *of attribute/score pairs and all evaluation functions *f*:

$\hat{h}\left(\left[\left(\hat{f}\times f\right)(x,y)|x\leftarrow xs,y\leftarrow ys\right]\right)=\hat{h}\left(\left[\left(\hat{f}\times f\right)(x,y)|x\leftarrow \hat{h}(xs),y\leftarrow \hat{h}(ys)\right]\right)\;\left(32\right)$

$\hat{h}$ (*xs *╫ *ys*) = $\hat{h}$ ($\hat{h}$(*xs*) ╫ $\hat{h}$(*ys*))     (33)

#### Proof

Equation 33 is simple to show. When solution *xs *and *ys *are classified separately, the objective function *h *is applied for each attribute twice, once on the corresponding sublist from *xs*, once on that from *ys*. Since by assumption *h *satisfies Bellman's Principle, it satisfies Equation 25 by itself, and applying *h *to the joined sublists for each attribute yields the same result.

To prove 32, we successively transform the LHS into the RHS.

$\begin{array}{l} \hat{h}\left(\left[(\hat{f}\times f)(u,v)|u\leftarrow xs,v\leftarrow ys\right]\right)=\\ \text{concat}\left[\left[\left(r^{j},w_{i}^{j}\right)\right]\right]\text{ where}\\ r^{j}\leftarrow \left\{\hat{f}(\hat{x},\hat{y})|(\hat{x},x)\leftarrow xs,(\hat{y},y)\leftarrow ys\right\}\\ w_{i}^{j}\leftarrow h\left[f(x,y)|(\hat{x},x)\leftarrow xs,(\hat{y},y)\leftarrow ys,\hat{f}(\hat{x},\hat{y})=r^{j}\right] \end{array}$

We first complete the proof under the additional assumption that the classification functions are confusion free – $\hat{f}$($\hat{a}$, $\hat{b}$) = $\hat{f}$($\hat{c}$, $\hat{d}$) implies $\hat{a}$ = $\hat{c}$ and $\hat{b}$ = $\hat{d}$. In this case, *r*^*j *^uniquely determines attributes *x *and y from which it arises via $\hat{x}$. The definition of $\hat{y}$ simplifies to

$w_{i}^{j}\leftarrow h([f(x,y)|(\hat{x},x)\leftarrow xs,(\hat{y},y)\leftarrow ys])$

Since classification is the identity on lists where all entries have the same attribute, and *h *satisfies 25, we can write

$w_{i}^{j}\leftarrow h\left(\left[f(x,y)|(\hat{x},x)\leftarrow \hat{h}(xs),(\hat{y},y)\leftarrow \hat{h}(ys)\right]\right)$

Together with the above definition of *r*^*j*^, we obtain

$\begin{array}{l} \text{concat}\left[\left[\left(r^{j},w_{i}^{j}\right)\right]\right]\text{ where}\\ r^{j}\leftarrow \left\{\hat{f}(\hat{x},\hat{y})|(\hat{x},x)\leftarrow xs,(\hat{y},y)\leftarrow ys\right\}\\ w_{i}^{j}\leftarrow h\left(\left[f(x,y)|(\hat{x},x)\leftarrow \hat{h}(xs),(\hat{y},y)\leftarrow \hat{h}(ys)\right]\right) \end{array}$

and by the definition of $\hat{h}$, this is

$\hat{h}\left(\left[\left(\hat{f}\times f\right)(u,v)|u\leftarrow \hat{h}(xs),v\leftarrow \hat{h}(ys)\right]\right)$

This completes the proof under our additional assumption of "no confusion". In the other case, the proof must consider all pairs of attributes ($\hat{a}$, $\hat{b}$) that can yield *r*^*j *^= $\hat{f}$($\hat{a}$, $\hat{b}$). As *h *distributes (by Equation 33) over the concatenation of these lists, the proof succeeds with an extra application of Equation 33.

### Why probabilistic shape analysis is expensive

Our algorithm computes accumulated probabilities (or Boltzman-weighted energies) for all shapes. Since the number of shapes grows exponentially with sequence length *n*, runtime is O (*n*^3^*P*^*n*^). It was determined empirically for shape level 5 in [16] that *P *≈ 1.1. *P *is somewhat larger for less abstract shapes, see Table 5. By contrast, MFE folding runs in O (*n*^3^), and computing the *k *best shape representatives takes O (*n*^3^·*k*), without incurring an exponential factor.

**Table 5:** Shape space sizes. Comparison of the shape space size for the 5 shape levels.

Shape level	1	2	3	4	5
Growth with *n*	1.26^*n*^	1.23^*n*^	1.16^*n*^	1.20^*n*^	1.10^*n*^

Since a small number of top-ranking shapes can be assumed to reveal all information relevant in applications, it seems plausible to compute probabilities for the best *k *shapes only, achieving runtime *O*(*kn*^3^) and avoiding the (albeit slow) exponential factor. Interestingly, this seems impossible – at least with the techniques used so far in simple and probabilistic shape analysis. The reason is that an objective function that maximizes accumulated shape probabilities would not satisfy Condition 2 (Eq. 25) of Bellman's Principle. An example suffices to demonstrate this.

Consider two alternative rules of structure formation, such as

sp <<< base ^~~~ ^closed ^~~~ ^base | | |

sr <<< base ^~~~ ^(bl <<< region ^~~~ ^closed) ^~~~ ^base

Let the first alternative return probabilities 0.3 and 0.2 for shapes [] and [[] []], and the second return 0.3 and 0.2 for [[] [] []] and [[] []]. Clearly, the optimal choice for both is 0.3, but as the optimal probabilities are derived for different shapes, they do not accumulate, and the overall best choice would be 0.2 + 0.2 = 0.4 for shape [[][]].

As optimal choice does not distribute over combinations of alternatives, Bellman's Principle is violated. However, accumulating scores for *all *shapes (without a choice of an optimal shape) is correct – at the extra cost of a slow exponential term in the runtime asymptotics.

This consideration applies to all schemes that accumulate scores over shapes. For example, if we score each structure simply by 1, the accumulated score is just the size of each shape's membership. This means that we have no polynomial time algorithm that determines the *k *largest shapes.

### Further implementation details

#### A non-ambiguous grammar with correct dangles

In the previous sections, we used the grammar attributed to Wuchty [9] to describe the general idea on how to calculate the probabilities of shapes. For expository reasons, the grammar presented has been simplified, while a faithful implementation of the current energy model requires more sophistication, in Wuchty's program as well as in ours.

A problem with Wuchty's full grammar is that it handles dangling bases in a simplified way, meaning that the grammar does not explicitly derive dangling bases. Instead, the free energy increment is added by the algebra irrespective of whether the bases are actually dangling. In general, this leads to lower free energies, which the developers see as an approximation for coaxial stacking. The effect of this inaccuracy would be that the partition function calculation and therefore the derived probabilities also show inaccuracies. We therefore modified this grammar in such a way that it handles dangling bases explicitly and unambiguously. This is achieved by imposing the following rules during grammar design, which apply to the external loop and to multiloops: (1) An unpaired base (singlestrand or dangling base) has to be followed by an unpaired base, either a singlestrand or a structural element with a dangling base. (2) A structural element without a dangling base on the 3'-end has to be followed by a structural element without a dangling base on the 5'-end. (3) We explicitly handle two structural elements with one unpaired base in between, to be able to decide which of the two possible dangling contributions is energetically favourable.

With the grammar designed in this way, we are able to derive all feasible secondary structures unambiguously with their correct energies and, therefore, also the correct partition function.

#### Practical efficiency

The nonambiguous, correct-dangle grammar has been implemented together with the algebras described in the previous section, yielding an algorithm for the exact calculation of probabilities for shapes. Application of this algorithm to various RNA sequences, of which some are shown in the Results Section, showed that the time and space requirements are quite moderate, allowing us to analyse sequences up to length 120 (with about 40 000 shapes) within 5 minutes on an Intel Xeon 2.8 GHz CPU.

#### Shape probabilities based on sampling

To be able to analyse longer sequences, we took up the idea of stochastic sampling introduced in [4]. This is achieved by changing the objective function *h *in the *bw *algebra from summation to picking one element randomly according to its Boltzmann weighted energy. This version can be used to draw samples (structures together with their shapes) of the structure space according to the Boltzmann distribution. Based on the shapes, the sample is partitioned into similarity classes and the frequency of each shape is computed. If the sample size is large enough, these shape frequencies come very close to the exact probabilities and can therefore be used instead.

We use our complete probabilistic shape analysis on moderate length sequences to evaluate how well the sample probabilities approximate the true ones with growing sample size, and relate the computational efforts required. The Results Section summarizes our observations using both algorithms.

### Availability and requirements

Project name: RNAshapes; Project home page: http://bibiserv.techfak.uni-bielefeld.de/rnashapes/. Operating systems: Source distribution and precompiled binary versions for Linux (i386), Solaris 8 (Sparc, i386), Microsoft Windows, MacOS X; See also [33].

## Authors' contributions

All authors contributed equally to this work. All authors read and approved the final manuscript.

**Table 6:** The full unambiguous grammar in EBNF notation. This is the full unambiguous grammar in EBNF notation. Note that dangling bases are not represented explicitly by a special terminal symbol, but as a 'base'. Their dangling property is accounted for by the derivation path, e.g. the secondary structure .( (...) ). for sequence 'ACCUAUGGG' will be derived as struct → left_dangle → edanglelr left_dangle → base initstem base left_dangle → base initstem base empty. The two unpaired bases in 'base initstem base' have been derived via ' edanglelr', which accounts for their dangling property. We do not give an explicit representation for dangling bases, as the need to derive them explicitly is due to the energy model and not to handling them as discrete structural elements, i.e. a dangling base is nothing more than an unpaired base next to a stem, but it has a non-positive energy contribution that cannot be neglected.

struct = left_dangle | noleft_dangle
left_dangle = base left_dangle |
edanglel base noleft_dangle |
edanglel (noleft_dangle | empty) |
edanglelr left_dangle |
empty
noleft_dangle = edangler left_dangle |
nodangle (noleft_dangle | empty) |
nodangle base noleft_dangle
edanglel = base initstem
edangler = initstem base
edanglelr = base initstem base
nodangle = initstem
initstem = closed
closed = stack | hairpin | multiloop | leftB | rightB | iloop
multiloop = base base base ml_comps1 base base |
base base base ml_comps2 base base |
base base ml_comps3 base base base |
base base ml_comps2 base base base |
base base base ml_comps4 base base base |
base base base ml_comps2 base base base |
base base base ml_comps1 base base base |
base base base ml_comps3 base base base |
base base ml_comps2 base base
ml_comps1 = block_dl no_dl_no_ss_end |
block_dlr dl_or_ss_left_no_ss_end |
block_dl base no_dl_no_ss_end
ml_comps2 = nodangle no_dl_no_ss_end |
edangler dl_or_ss_left_no_ss_end |
nodangle base no_dl_no_ss_end
ml_comps3 = nodangle no_dl_ss_end |
nodangle base no_dl_ss_end
ml_comps4 = block_dl no_dl_ss_end |
block_dlr dl_or_ss_left_ss_end |
block_dl base no_dl_ss_end
block_dl = region edanglel |
edanglel
block_dlr = region edanglelr |
edanglelr
no_dl_no_ss_end = ml_comps2 |
nodangle
dl_or_ss_left_no_ss_end = ml_comps1 |
block_dl
no_dl_ss_end = ml_comps3 |
edangler |
edangler region
dl_or_ss_left_ss_end = ml_comps4 |
block_dlr |
block_dlr region |
stack = base closed base
hairpin = base base region base base
leftB = base base region initstem base base
rightB = base base initstem region base base
iloop = base base region closed region base base
base = ' A ' | ' C ' | ' G ' | ' U '
region = base |
base region

## References

[B1] Zuker M Stiegler P Optimal computer folding of large RNA sequences using thermodynamics and auxiliary information Nucleic Acids Res 1981 9 133 148 6163133 10.1093/nar/9.1.133 PMC326673

[B2] Mathews D Sabina J Zuker M Turner H Expanded Sequence Dependence of Thermodynamic Parameters Provides Robust Prediction of RNA Secondary Structure J Mol Biol 1999 288 911 940 10.1006/jmbi.1999.2700 10329189

[B3] Doshi K Cannone J Cobaugh C Gutell R Evaluation of the suitability of free-energy minimization using nearest-neighbor energy parameters for RNA secondary structure prediction BMC Bioinformatics 2004 5 105 126 10.1186/1471-2105-5-105 15296519 PMC514602

[B4] Ding Y Lawrence CE A statistical sampling algorithm for RNA secondary structure prediction Nucleic Acids Res 2003 31 7280 7301 10.1093/nar/gkg938 14654704 PMC297010

[B5] Meyer I Miklos I Co-transcriptional folding is encoded within RNA genes BMC Mol Biol 2004 5 10 19 10.1186/1471-2199-5-10 15298702 PMC514895

[B6] Zuker M On Finding All Suboptimal Foldings of an RNA Molecule Science 1989 244 48 52 2468181 10.1126/science.2468181

[B7] Zuker M Mathews D Turner D Barciszewski J, Clark B Algorithms and Thermodynamics for RNA Secondary Structure Prediction: A Practical Guide RNA Biochemistry and Biotechnology 1999 NATO ASI Series, Kluwer Academic Publishers

[B8] Zuker M Mfold web server for nucleic acid folding and hybridization prediction Nucl Acids Res 2003 31 3406 3415 10.1093/nar/gkg595 12824337 PMC169194

[B9] Wuchty S Fontana W Hofacker I Schuster P Complete suboptimal folding of RNA and the stability of secondary structures Biopolymers 1999 49 145 165 10.1002/(SICI)1097-0282(199902)49:2<145::AID-BIP4>3.0.CO;2-G 10070264

[B10] Hofacker I Fontana W Stadler P Bonhoeffer L Tacker M Schuster P Fast Folding and Comparison of RNA Secondary Structures (The Vienna RNA Package) Chemical Monthly 1994 125 167 188 10.1007/BF00818163

[B11] Smith T Waterman M Identification of common molecular subsequences J Mol Biol 1981 147 195 197 10.1016/0022-2836(81)90087-5 7265238

[B12] McCaskill J The equilibrium Partition Function and Base Pair Binding Probabilities for RNA Secondary Structure Biopolymers 1990 29 1105 1119 10.1002/bip.360290621 1695107

[B13] Giegerich R Haase D Rehmsmeier M Prediction and visualization of structural switches in RNA Pac Symp Biocomput 1999 126 137 10380191 10.1142/9789814447300_0013

[B14] Voss B Meyer C Giegerich R Evaluating the Predictability of Conformational Switching in RNA Bioinformatics 2004 20 1573 1582 10.1093/bioinformatics/bth129 14962925

[B15] Flamm C Hofacker I Stadler P Wolfinger M Barrier Trees of Degenerate Landscapes Z Phys Chem 2002 216 155 173

[B16] Giegerich R Voss B Rehmsmeier M Abstract Shapes of RNA NAR 2004 32 16 4843 4851 10.1093/nar/gkh779 15371549 PMC519098

[B17] Giegerich R Explaining and Controlling Ambiguity in Dynamic Programming Proc Combinatorial Pattern Matching 2000 46 59

[B18] Dowell R Eddy S Evaluation of several lightweight stochastic context-free grammars for RNA secondary structure prediction BMC Bioinformatics 2004 5 71 10.1186/1471-2105-5-71 15180907 PMC442121

[B19] Reeder J Steffen P Giegerich R Effective ambiguity checking in biosequence analysis BMC Bioinformatics 2005 6 153 15967024 10.1186/1471-2105-6-153 PMC1215473

[B20] Giegerich R Meyer C Steffen P A Discipline of Dynamic Programming over Sequence Data Science of Computer Programming 2004 51 215 263 10.1016/j.scico.2003.12.005

[B21] Walter A Turner D Kim J Lyttle M Müller P Mathews D Zuker M Coaxial Stacking of Helixes Enhances Binding of Oligoribonucleotides and Improves Predictions of RNA Folding Proc Natl Acad Sci USA 1994 91 9218 9222 7524072 10.1073/pnas.91.20.9218 PMC44783

[B22] Chen G Znosko B Jiao X Turner D Factors Affecting Thermodynamic Stabilities of RNA 3 × 3 Internal Loops Biochemistry 2004 43 12865 12876 10.1021/bi049168d 15461459

[B23] Schilling O Langbein I Müller M Schmalisch M Stülke J A protein-dependent riboswitch controlling *ptsGHI *operon expression in *Bacillus subtilis*: RNA structure rather than sequence provides interaction specificity Nucl Acids Res 2004 32 2853 2864 10.1093/nar/gkh611 15155854 PMC419612

[B24] Bonnet E Wuyts J Rouzé P Van de Peer Y Evidence that microRNA precursors, unlike other non-coding RNAs, have lower folding free energies than random sequences Bioinformatics 2004 20 15217813 10.1093/bioinformatics/bth374

[B25] Olsen P Ambros V The lin-4 regulatory RNA controls developmental timing in Caenorhabditis elegans by blocking LIN-14 protein synthesis after the initiation of translation Dev Biol 1999 216 671 680 10.1006/dbio.1999.9523 10642801

[B26] Ding Y Chan C Lawrence CE RNA secondary structure prediction by centroids in a Boltzmann weighted ensemble RNA 2005 11 8 1157 66 10.1261/rna.2500605 16043502 PMC1370799

[B27] Le S Chen J Maizel J Structure and methods: Human Genome Initiative and DNA recombination, Efficient searches for unusual folding regions in RNA sequences 1990 Adenine Press, Schenectady, NY 127 136

[B28] Seffens W Digby D mRNAs have greater negative folding free energies than shuffled or codon choice randomized sequences Nucl Acids Res 1999 27 1578 1584 10.1093/nar/27.7.1578 10075987 PMC148359

[B29] Workman C Krogh A No evidence that mRNAs have lower folding free energies than random sequences with the same dinucleotide distribution Nucl Acids Res 1999 27 24 4816 4822 10.1093/nar/27.24.4816 10572183 PMC148783

[B30] Rivas E Eddy S Secondary structure alone is generally not statistically significant for the detection of noncoding RNAs Bioinformatics 2000 16 583 605 10.1093/bioinformatics/16.7.583 11038329

[B31] Clote P Ferré F Kranakis E Krizanc D Structural RNA has lower folding energy than random RNA of the same dinucleotide frequency RNA 2005 11 578 591 10.1261/rna.7220505 15840812 PMC1370746

[B32] Bellmann R Dynamic Programming 1957 Princeton, NJ: Princeton University Press

[B33] Steffen P Voß B Rehmsmeier M Reeder J Giegerich R RNAshapes: an integrated RNA analysis package based on abstract shapes Bioinformatics 2006 22 4 500 503 10.1093/bioinformatics/btk010 16357029

